# Layered double hydroxide/sepiolite hybrid nanoarchitectures for the controlled release of herbicides

**DOI:** 10.3762/bjnano.10.163

**Published:** 2019-08-09

**Authors:** Ediana Paula Rebitski, Margarita Darder, Pilar Aranda

**Affiliations:** 1 Instituto de Ciencia de Materiales de Madrid, CSIC, c/ Sor Juana Inés de la Cruz 3, Cantoblanco, 28049 Madrid, Spain

**Keywords:** controlled release, hybrid nanoarchitectures, layered double hydroxides, 2-methyl-4-chlorophenoxyacetic acid (MCPA), nanoarchitectonics, sepiolite

## Abstract

In this work, organic–inorganic hybrid nanoarchitectures were prepared in a single coprecipitation step by assembling magnesium–aluminum layered double hydroxides (MgAl-LDH) and a sepiolite fibrous clay, with the simultaneous encapsulation of the herbicide 2-methyl-4-chlorophenoxyacetic acid (MCPA) as the MgAl-LDH retains its ion exchange properties. The synthetic procedure was advantageous in comparison to the incorporation of MCPA by ion exchange after the formation of the LDH/sepiolite nanoarchitecture in a previous step, as it was less time consuming and gave rise to a higher loading of MCPA. The resulting MCPA-LDH/sepiolite nanoarchitectures were characterized by various physicochemical techniques (XRD, FTIR and ^29^Si NMR spectroscopies, CHN analysis and SEM) that revealed interactions of LDH with the sepiolite fibers through the silanol groups present on the outer surface of sepiolite, together with the intercalation of MCPA in the LDH confirmed by the increase in the basal spacing from 0.77 nm for the pristine LDH to 2.32 nm for the prepared materials. The amount of herbicide incorporated in the hybrid nanoarchitectures prepared by the single-step coprecipitation method surpassed the CEC of LDH (ca. 330 mEq/100 g), with values reaching 445 mEq/100 g LDH for certain compositions. This suggests a synergy between the inorganic solids that allows the nanoarchitecture to exhibit better adsorption properties than the separate components. Additionally, in the release assays, the herbicide incorporated in the hybrid nanoarchitectures could be completely released, which confirms its suitability for agricultural applications. In order to achieve a more controlled release of the herbicide and to act for several days on the surface of the soil, the hybrid nanoarchitectures were encapsulated in a biopolymer matrix of alginate/zein and shaped into spheres. In in vitro tests carried out in bidistilled water, a continuous release of MCPA from the bionanocomposite beads was achieved for more than a week, while the non-encapsulated materials released the 100% of MCPA in 48 h. Besides, the encapsulation may allow for better handling and transport of the herbicide.

## Introduction

Nanoarchitectonics is a definition attributed to the development of materials with new functionalities based on a controlled arrangement of nanoscale structural units through their mutual interactions [1]. The term “nanoarchitectonics” coined at the "MANA" research center (Nanoscale Materials Division of the National Institute of Materials Science (NIMS) in Japan) is based on five main concepts: i) controlled self-organization, ii) chemical nanomanipulation, iii) field-induced material control, iv) new manipulations of atoms and molecules, and v) theoretical modeling and design [1–2]. Based on these premises a large number of nanoarchitectonic materials have been prepared including mesoporous solids, self-organized block-copolymers, supramolecular materials, and macromolecular systems of DNA and cells [2–5]. In this context, clay-based nanoarchitectonic materials have been developed over the years, starting from classical pillared clays and porous clay heterostructures (PCH) to more innovative materials involving the assembly of different types of nanoparticles and other species, and clays of different origin and morphology [6–11]. There are diverse methodologies and synthesis strategies to provide new functionalities to clays. Particularly useful for constructing nanoarchitectures is the use of organic–inorganic interphases as those provided by organoclays [12]. Besides typical 2D layered clays, fibrous (sepiolite, palygorskite) and tubular (halloysite, imogolite) clays are attracting growing interest in the development of a large variety of functional nanomaterials and nanocomposites for application in diverse fields [13–15].

**Figure 1:**
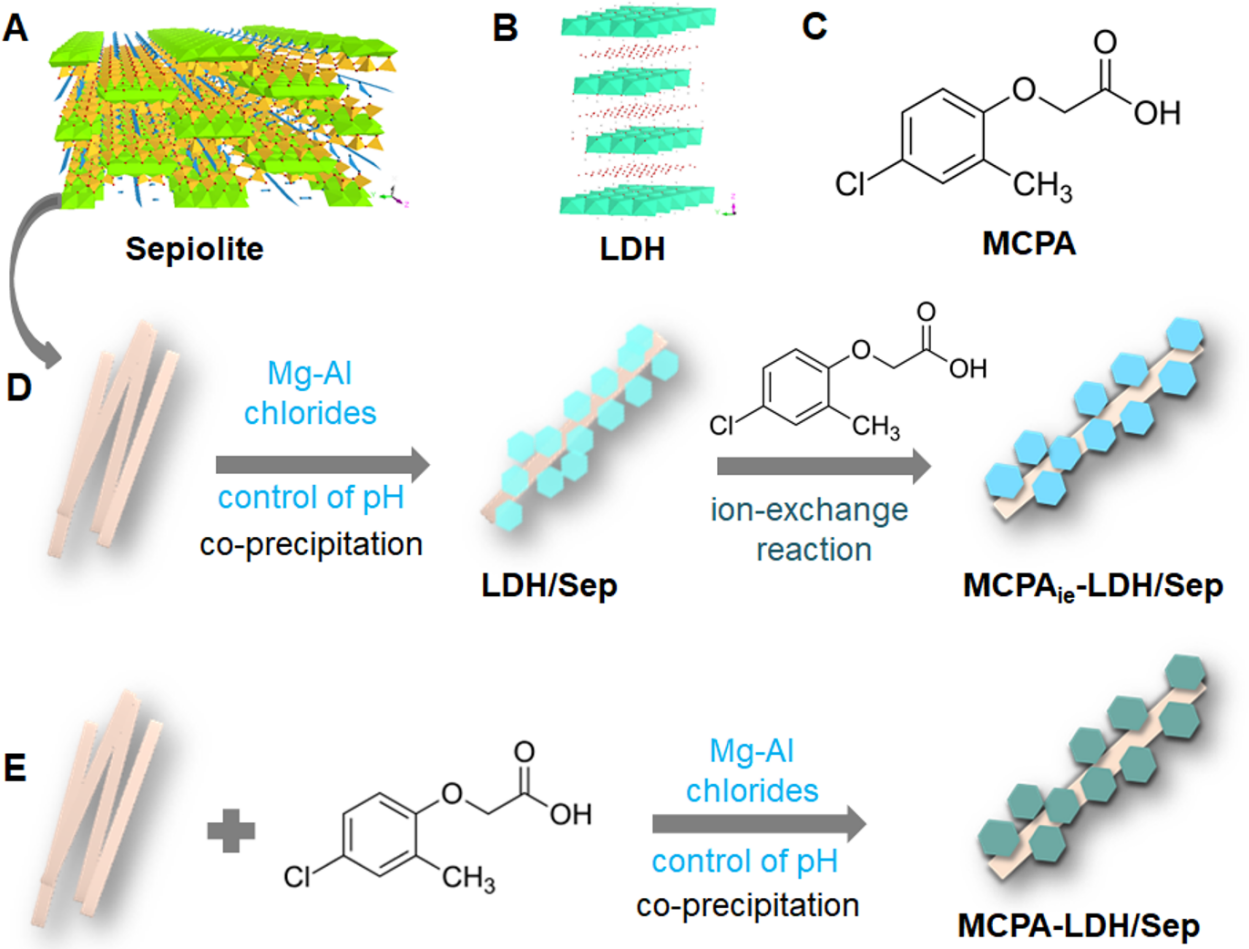
Schematic representations of (A) sepiolite and (B) layered double hydroxide structures, (C) molecular structure of 2-methyl-4-chlorophenoxyacetic acid (MCPA), and schemes of the synthesis of the hybrid MCPA-LDH/sepiolite nanoarchitectures by (D) ion exchange and (E) one-step coprecipitation.

Sepiolite (Figure 1A) is a natural hydrated magnesium silicate with the ideal formula [Si_12_O_30_Mg_8_(OH,F)_4_](H_2_O)_4_·8H_2_O [16–17], which exhibits high surface area and adsorption capacity due to the presence of silanol groups on the external surface of the clay fibers. These ≡SiOH groups are arranged regularly along the structural edges of the fiber, being advantageous to produce functional nanoarchitectures. Thus, in recent years the number of publications related to the assembly of different types of nanoparticulated solids (e.g., metals, metal oxides, and graphene) and sepiolite or palygorskite has increased, yielding nanoplatforms useful in a large number of applications from catalysis, environmental remediation, energy production and storage to biomedicine [14,18]. The co-assembly of particles can be reached through several methods, from the direct assembly of the clay to diverse nanoparticulated solids to the in situ generation of nanoparticles in the presence of the clay [14]. One of the key points in these strategies is to reach a good disaggregation of the fibrous particles to favor the exposition of the clay surface for the assembly with other particles, either present in the medium or in the process of growing. Examples are the direct assembly of carbon nanotubes and sepiolite under ultrasonic irradiation [19] and the generation of layered titanosilicates in the presence of sepiolite [20]. In this context, the use of organic–inorganic interphases has proved highly effective to facilitate the co-assembly process, which favors the formation of more homogeneous and, in general, better organized nanoarchitectures [12].

Layered double hydroxides (LDH), often called anionic clays, are 2D solids of the general formula [M(II)_1−_*_x_*M(III)*_x_*(OH)_2_]*^x^*^+^(A*^n^*^−^)*_x_*_/_*_n_*·*m*H_2_O (Figure 1B), consisting of positively charged brucite-like layers that are balanced with anions and water molecules in the interlayer space [21]. Although there also exists in nature a Mg–Al layered double hydroxide, namely hydrotalcite, LDH materials can be easily prepared by coprecipitation from metal solutions at a controlled pH value. This procedure and other protocols of synthesis together with the possibility to stabilize solids involving a large variety of metal ions have provided a large variety of LDH compounds of interest in numerous applications as adsorbents of anionic pollutants, catalysts, additive of polymers, as components in diverse electrochemical devices (such as supercapacitors, sensors, and biosensors), in drug delivery and controlled-release formulations, or in non-viral gene transfection [21–26]. The fact that the stability of LDH varies with the pH value has proved advantageous in some of the above mentioned applications, in particular, for uses as host substrate in the immobilization of active species (e.g., drugs, pesticides, and DNA) for controlled-delivery applications [27–29]. LDH have been also used in the construction of different types of nanoarchitectonic materials. The used strategies included wet impregnation and layer-by-layer approaches to produce diverse type of multilayer heterostructures, e.g., ZnCr-LDH/TiO_2_ films [30], in situ formation of the LDH in presence of other nanoparticles, e.g., sepiolite [31], and reconstruction of the LDH from parent “layered double oxides” in the presence of diverse species, e.g., silica nanoparticles [32].

Nanoarchitectonic materials involving the growth of LDH nanoparticles in the presence of fibrous clay silicates were patented several years ago [33]. Direct co-assembly of already formed particles of each component does not produce true nanoarchitectonic materials. Hence, it is necessary to grow the LDH in the presence of the fibrous clay [31]. In fact, the presence of silanol groups along the external surface of the silicate fibers act as anchoring points at which the LDH grows, forming LDH particles with their characteristic sandrose structure surrounding the clay fibers [31]. The resulting materials may show dual ion exchange behavior due to the anion and cation exchange properties of LDH and sepiolite components, respectively. This type of nanoarchitectonic materials could be of interest as adsorbents for the removal of pollutants from water, for instance dyes [31] and, As(III) and As(V) species [34]. Moreover, they could be used as precursors for supported metal-oxide nanoparticles that could be of interest in catalysis [31]. MgAl-LDH/sepiolite nanoarchitectures have been also satisfactorily tested as nanofiller in Nafion membranes for fuel-cell applications [35]. With these premises, the current aim is to ascertain if it is possible to develop organic–inorganic hybrid materials using LDH-sepiolite nanoarchitectonic materials, as the presence of an organic counterpart could be of interest for introducing additional functionalities. Thus, in this first work, we have explored the incorporation of an anionic molecule, the herbicide 2-methyl-4-chlorophenoxyacetic acid (MCPA, Figure 1C), as it is expected to easily associate with the LDH. The resulting materials showed MCPA release properties that allow for the application of these systems for the controlled delivery of this herbicide. Hybrid nanoarchitectures were prepared profiting from the anion exchange properties of the MgAl-LDH/sepiolite and also by coprecipitation of the MgAl-LDH in the presence of an aqueous dispersion of sepiolite in which MCPA was also present. Differences in composition, structure and release behavior between the developed hybrid nanoarchitectures prepared by the two methods were examined and analyzed. In view to apply these materials in agriculture, the efficiency of formulations based on the hybrid nanoarchitectures was explored in in vitro tests of MCPA release, confirming the improvement of retention properties. For a better control in the MCPA release, the hybrid nanoarchitectures were also combined with mixtures of alginate–zein biopolymers [36] to improve the retention properties.

## Results and Discussion

### MCPA-LDH/sepiolite hybrid nanoarchitectures

**Figure 2:**
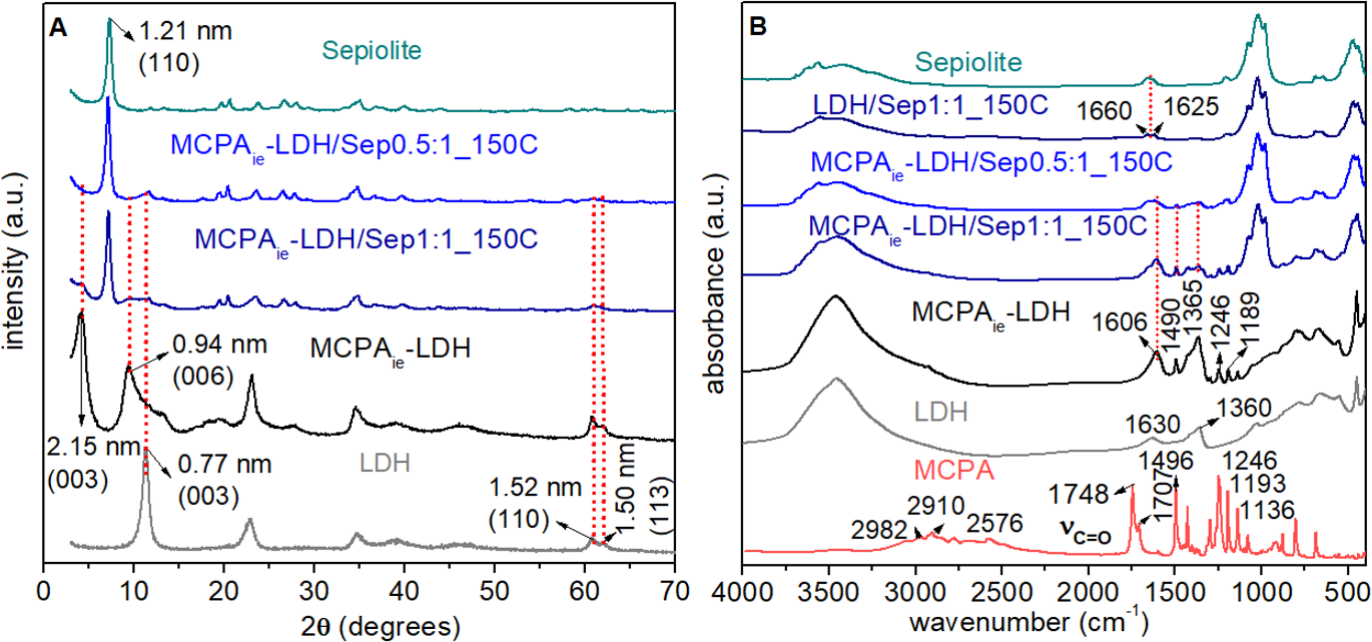
(A) XRD patterns and (B) FTIR spectra of individual components (sepiolite, LDH, and MCPA), MCPA_ie_-LDH intercalation compound and the neat LDH/Sep and MCPA_ie_-LDH/Sep hybrid nanoarchitectures.

The preparation of MgAl-LDH/sepiolite (LDH/Sep) hybrid nanoarchitectures was firstly achieved by ion exchange of MCPA herbicide anions with the chloride ions present in LDH/Sep nanoarchitectures previously prepared following the protocol reported elsewhere by Gomez-Avilés et al. [31] (Figure 1D). XRD patterns (Figure 2A) of both nanoarchitectures, as prepared and after the ion exchange reaction, showed the most intense peaks in the patterns of the pure sepiolite and the LDH. The differences in the position of the most intense peak ascribed to the LDH in the neat nanoarchitectures and most of the hybrid nanoarchitectures confirm the intercalation of MCPA in the LDH supported on the sepiolite fibers. The *d*(003) reflection is shifted towards lower 2θ angles, resulting from an increase of the basal spacing from 0.77 to 2.15 nm, with values similar to those observed when MCPA is intercalated in the LDH [37–38]. FTIR spectra (Figure 2B) shows bands ascribed to the organic component in all of the hybrid nanoarchitectures, although, as occurs in the MCPA_ie_-LDH intercalation compound, interactions with the inorganic substrate modified the position of the bands. This affects specially to the very intense bands at 1748 and 1707 cm^−1^ assigned to the ν_C=O_ vibration modes of the carboxylic group of MCPA, which are not observed in the spectra of both the MCPA_ie_-LDH and the MCPA_ie_-LDH/Sep1:1_150C hybrids (Figure 2B). They are shifted towards lower wavenumbers expected at around 1610 cm^−1^ (symmetric and asymmetric stretching vibration of ionized COO^−^ groups) [39] as the carboxylic group should be present as carboxylate. In fact, the spectra show a large band in the range of 1630–1600 cm^−1^ due to the overlap of such bands with the one ascribed to δ_HOH_ vibration modes of water molecules adsorbed on the inorganic solids that appear at around 1630 cm^−1^ [40]. In addition around 1360 and 1365 cm^−1^, in the initial LDH and in the MCPA_ie_-LDH, a possible contamination with carbonate ions during the preparation of the materials is observed (Figure 2B) [41].

The amounts of MCPA present in each nanoarchitecture were determined by elemental chemical analysis (CHN) and expressed in relation to the amount of LDH present in the nanoarchitectures (Table 1). The expected anion exchange capacity (AEC) of the LDH is around 330 mEq/100 g LDH, and so the content in MCPA in the MCPA_ie_-LDH intercalation compound suggests the ion exchange process is incomplete in the adopted experimental conditions. The content of MCPA in the MCPA_ie_-LDH/Sep1:1_150C hybrid nanoarchitecture is similar to that of the MCPA_ie_-LDH hybrid. However, the expected content for a complete ion exchange is reached in the MCPA_ie_-LDH/Sep0.5:1_150C material. This effect could be ascribed to a lower degree of agglomeration of the LDH particles grown on the sepiolite fibers in the nanoarchitecture with lower content in LDH, which may favor a faster ion exchange reaction. In fact, in MCPA_ie_-LDH/Sep0.3:1_150C, in which the sepiolite fibers are less covered, the amount of MCPA surpassed the ion exchange capacity of the LDH. This fact might be related to interaction of MCPA anions with hydrogen atoms of the silanol groups on the surface of sepiolite, acting as new points for MCPA adsorption. In fact, sepiolite may adsorb MCPA up to approx. 100 mg of MCPA per gram of sepiolite (see Figure S1, Supporting Information File 1).

**Table 1:** Amounts of MCPA in mEq/100 g of the hybrid and materials prepared by the ion exchange method.

sample	LDH/Sep real ratio	MCPA-LDH/Sep real ratio	mEq of MCPA/100 g of LDH

MCPA_ie-_LDH	—	—	278
MCPA_ie_-LDH/Sep1:1_150C	0.94:1	0.90:1	269
MCPA_ie_-LDH/Sep0.5:1_150C	0.47:1	0.45:1	325
MCPA_ie_-LDH/Sep0.3:1_150C	0.28:1	0.27:1	452

**Figure 3:**
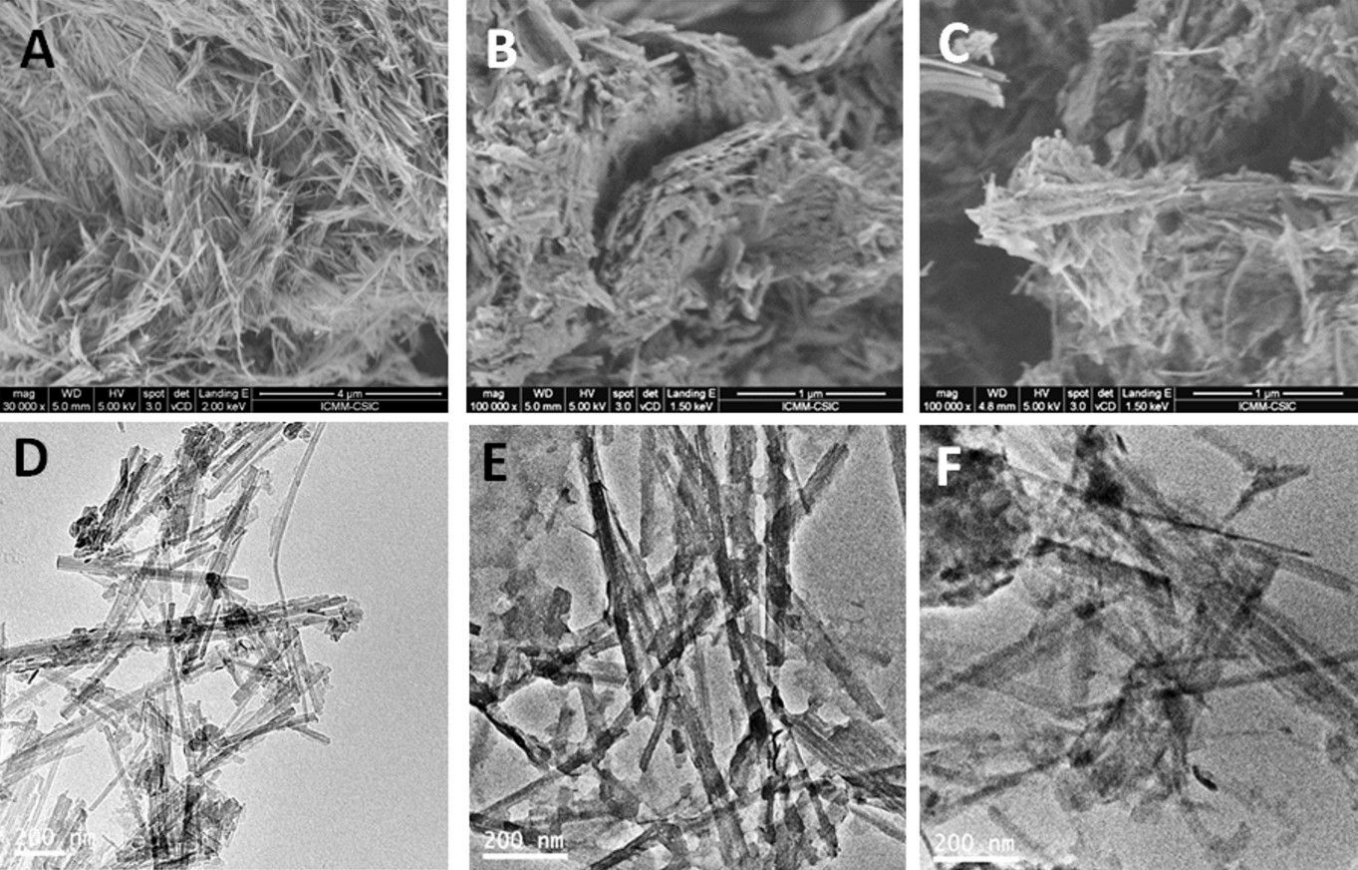
FE-SEM images of (A) sepiolite, (B) LDH/Sep1:1_150C and (C) MCPA_ie_-LDH/Sep1:1_150C nanoarchitectures; TEM images of (D) LDH/Sep0.5:1_150C, and the LDH/Sep1:1_150C nanoarchitecture (E) before and (F) after the ion exchange treatment with MCPA.

Figure 3 shows images obtained by FE-SEM and TEM from the neat sepiolite and from the hybrid nanoarchitectures. The FE-SEM images show that the sepiolite fibers appear covered and compacted after the coprecipitation process to produce the corresponding nanoarchitecture. The aspect of the as prepared material and the material recovered after the intercalation of MCPA in the LDH component does not vary significantly. This fact is confirmed by TEM (Figure 3E,F), where it is possible to distinguish the presence of small flat particles attached to the fibers in both nanoarchitectures. These images also confirm that the ion exchange treatment is in fact a topotactic intercalation process that does not affect the nature of the LDH/sepiolite nanoarchitecture, confirming also the stability of this type of materials. In addition, FE-SEM and TEM images (Figure S2, Supporting Information File 1) show that the starting LDH and the MCPA_ie_-LDH material exhibit small and uniform particles around 100 nm in diameter.

### MCPA-LDH/sepiolite hybrid nanoarchitectures prepared via coprecipitation

MCPA-LDH intercalation compounds can be also produced by coprecipitation of the LDH in the presence of sepiolite and MCPA. The high pH value during the formation of MgAl-LDH facilitates the incorporation of MCPA as charge-compensating interlayer anion. The amount of adsorbed MCPA varies with the LDH/sepiolite ratio in the hybrid nanoarchitecture. Unexpectedly, large amounts of MCPA are taken up when the amount of LDH is reduced (Table 2). Moreover, it seems that the presence of large amounts of MCPA is accompanied by a lower yield of assembled LDH particles in the nanoarchitecture, which can be reduced to half for nanoarchitectures with a theoretical LDH/sepiolite composition of 0.5:1. In most of the prepared hybrid nanoarchitectures, the amount of MCPA exceeds the anionic exchange capacity of the LDH (ca. 330 mEq/100 g), which suggests that a part of the MCPA is adsorbed by another mechanism, perhaps on the external surface of the sepiolite clay or in interaction with the clay and the LDH particles. We have confirmed that at the pH value used in the synthesis process there is no precipitation of Al-MCPA or Mg-MCPA salts, although at lower pH values it is possible to produce precipitates in the presence of Al^3+^ ions. As reported in previous studies [42] and mentioned above, it has been demonstrated that sepiolite does not absorb large amounts of MCPA. However, we have observed that the adsorption of MCPA on sepiolite increases in the presence of Mg^2+^ and Al^3+^ salts at pH values below those required for the precipitation of the LDH (Table S1, Supporting Information File 1). This might occur during the coprecipitation of the LDH in the presence of MCPA. Given that this synthesis involves an organic molecule, the hybrid nanoarchitectures were heat-treated at 150 °C as in [31], and also at a lower temperature of 60 °C. Both thermal treatments resulted in similar materials, showing that lower temperatures could be used when less stable organic molecules are involved.

**Table 2:** LDH yield, LDH/Sep ratio and amount of MCPA incorporated in the hybrid nanoarchitectures prepared via coprecipitation.

sample	LDH yield (%)	LDH/Sep real ratio	mEq MCPA/100 g LDH

MCPA-LDH	89.7	—	303
MCPA-DH/Sep2:1_60C	84.0	1.68:1	336
MCPA-LDH/Sep2:1_150C	82.0	1.64:1	356
MCPA-LDH/Sep1:1_60C	81.0	0.81:1	385
MCPA-LDH/Sep1:1_150C	78.0	0.78:1	421
MCPA-LDH/Sep0.5:1_60C	78.0	0.39:1	433
MCPA-LDH/Sep0.5:1_150C	77.0	0.38:1	445
MCPA-LDH/Sep0.3:1_60C	54.4	0.18:1	1266
MCPA-LDH/Sep0.3:1_150C	41.4	0.19:1	1180

**Figure 4:**
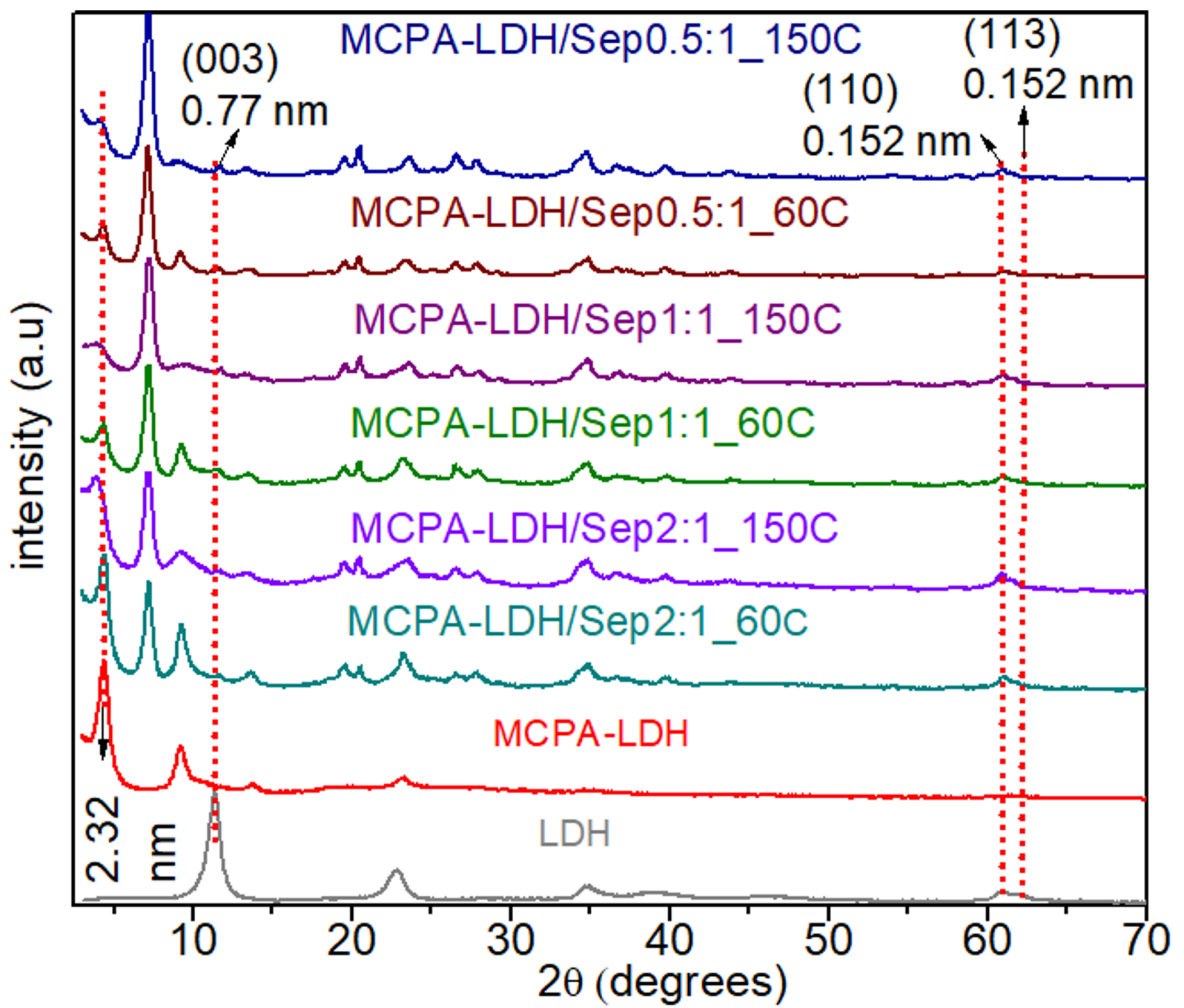
XRD patterns of hybrid nanoarchitectures prepared by coprecipitation of MgAl-LDH in the presence of sepiolite and MCPA at different theoretical LDH/sepiolite ratios (*x*:1).

XRD patterns of the hybrid nanoarchitectures (Figure 4) confirmed that in all cases MCPA is intercalated in the interlayer space of the coprecipitated LDH, as indicated by the presence of the *d*(003) reflection peak characteristic of the LDH structure at a 2θ angle around 4.5°. From that reflection, basal spacing values of 2.32 nm are deduced in the LDH present in the hybrid nanoarchitectures, which is similar to that determined in MCPA-LDH intercalation compounds prepared by both ion exchange and coprecipitation. The structure of sepiolite is maintained in all samples, independent of the proportion of LDH, while the most intense peak of the LDH decreased in intensity at the same time that the proportion of LDH/sepiolite is lowered. In addition, the LDH peaks *d*(110) and *d*(113) are observed in all the nanoarchitectures formed, confirming the formation of the LDH structure for all the studied LDH/sepiolite ratios.

**Figure 5:**
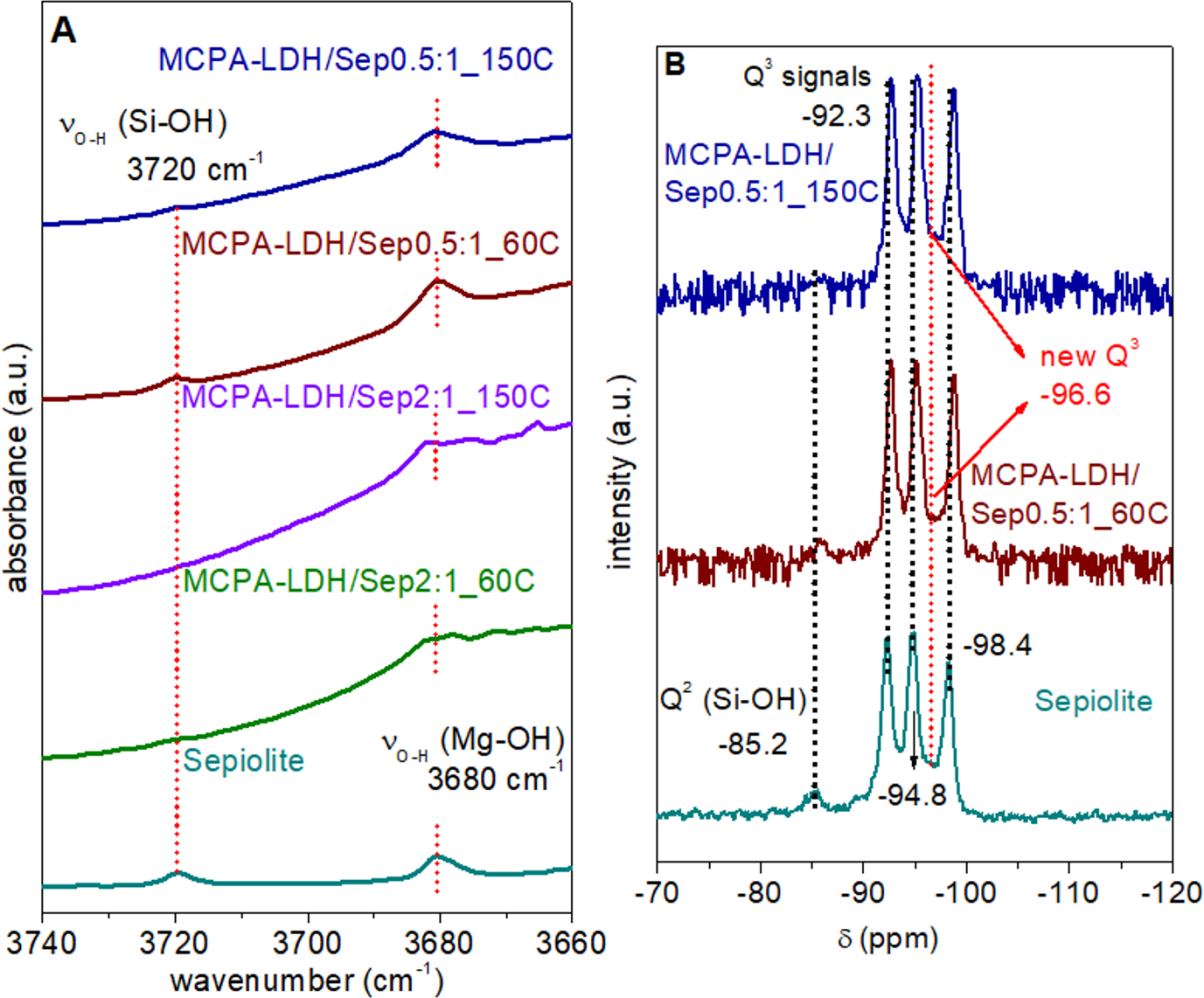
(A) FTIR (3800 to 3600 cm^−1^ region) and (B) ^29^Si MAS NMR spectra of neat sepiolite and hybrid nanoarchitectures prepared by coprecipitation of the MgAl-LDH in the presence of sepiolite and MCPA at different theoretical LDH/sepiolite ratios (*x*:1).

The formation of true hybrid nanoarchitectures was confirmed by infrared and NMR spectroscopy. For this purpose, the spectral region of the OH vibration bands of the Si–OH and Mg–OH groups was analyzed in detail. These bands appear at approximately 3720 and 3680 cm^−1^, respectively, in the IR spectrum of pure sepiolite [43]. The band attributed to the OH vibration of the Mg–OH groups is observed in the hybrid nanoarchitectures with apparently the same intensity. In contrast, the intensity of the band at 3720 cm^−1^ associated with vibrations of Si–OH groups is attenuated in the hybrid nanoarchitectures, indicating that part of those silanol groups are in interaction with other species as observed in other modifications of sepiolite [44–47]. This perturbation originates from hydrogen interactions between the silanol groups of the silicate and LDH particles, inducing a shift of the associated IR band towards lower frequencies. In fact, the band practically becomes imperceptible, mainly in the MCPA-LDH/Sep hybrid nanoarchitectures after thermal treatment at the highest temperature (Figure 5A). Also, this band is not observed in samples containing the highest proportions of LDH with respect to sepiolite, where the LDH particles may be completely covering the sepiolite fibers. The chemical interactions between the LDH and sepiolite components in the LDH/Sep hybrid nanoarchitectures prepared by coprecipitation were also corroborated by ^29^Si MAS NMR (Figure 5B). As previously reported [31], the spectrum of MgAl-LDH/Sep is different from that of pure sepiolite. The spectrum of neat sepiolite shows three Q^3^ signals and one Q^2^ signal. The latter one is associated with the silanol groups [48]. In the spectra of the MCPA-LDH/Sep0.5:1_60C and MCPA-LDH/Sep0.5:1_150C hybrid nanoarchitectures the Q^3^ signals are slightly shifted to values around −92.3, −94.8 and −98.4 ppm, while the Q^2^ signal is practically not detected (Figure 5B). In addition, a new Q^3^ signal, is observed at −96.6 ppm, which could be associated with a new type of Si environment coming from the condensation of the silanol –OH groups on the surface of the sepiolite fibers with the hydroxy groups of the co-precipitated LDH particles, as previously reported for neat LDH/sepiolite nanoarchitectures [31]. The small differences observed in the FTIR and NMR spectra of hybrid nanoarchitectures prepared by consolidation at 60 and 150 °C indicate the high stability of the prepared materials after both thermal treatments. This confirms the possibility of consolidating the hybrid nanoarchitectures at mild temperatures below 150 °C. FTIR spectroscopy also confirms the incorporation of MCPA through interaction with the LDH (Figure S3, Supporting Information File 1), as discussed for the hybrid nanoarchitectures prepared by ion exchange.

**Figure 6:**
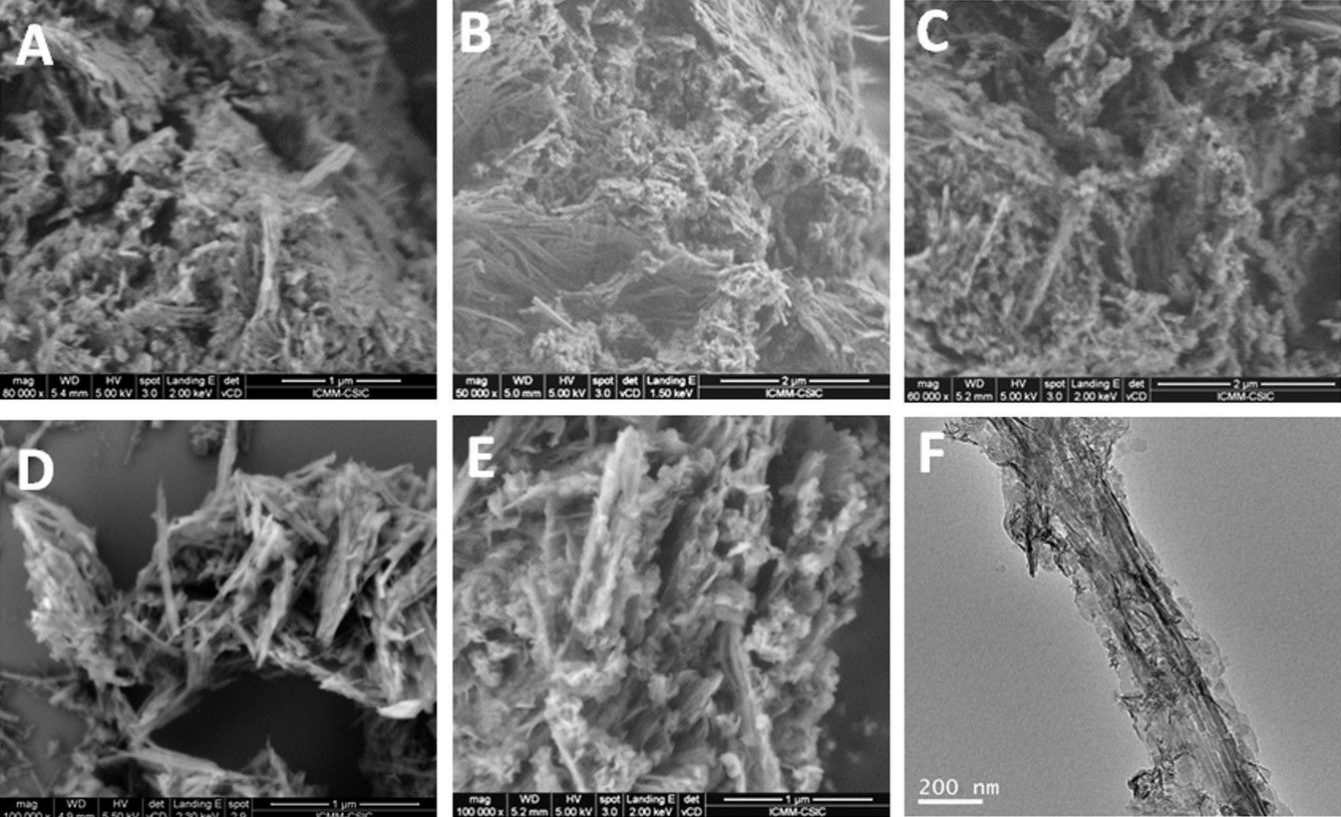
FE-SEM images of (A) MCPA-LDH/Sep2:1_150C, (B) MCPA-LDH/Sep1:1_150C, (C) MCPA-LDH/Sep0.5:1_60C, (D) LDH/Sep0.5:1_150C, and (E) MCPA-LDH/Sep0.5:1_150C hybrid nanoarchitectures prepared by coprecipitation from different LDH/sepiolite ratios; (F) TEM image of the MCPA-LDH/Sep0.5:1_60C hybrid nanoarchitecture.

The FE-SEM images of the MCPA-LDH/Sep hybrid nanoarchitectures (Figure 6) confirm that sepiolite fibers are covered by LDH nanoparticles, which are more agglomerated in the hybrid nanoarchitectures containing higher amounts of LDH. In the structures with lower LDH content, the layered solid grows in particles of smaller size and TEM images clearly confirm that they remain attached to the silicate fibers (Figure 6F).

### In vitro release of MCPA in water

**Figure 7:**
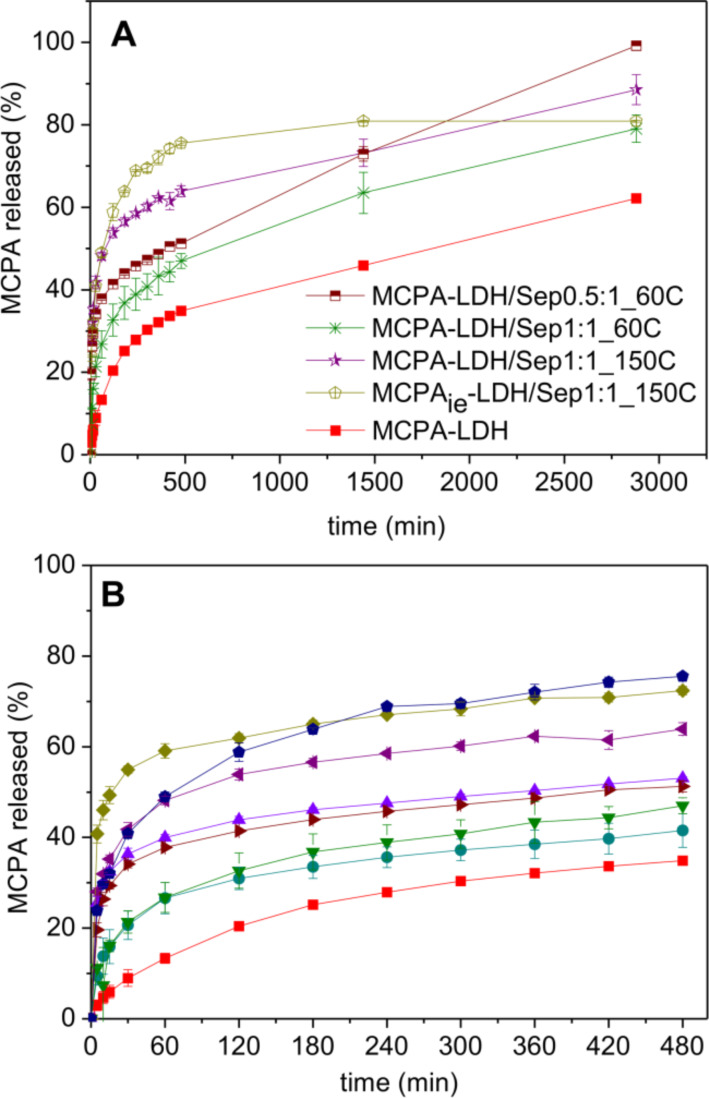
(A) In vitro release of MCPA from the hybrid formulations in deionized water (pH approx. 5.5), and (B) zoom of the same graph showing the release behavior in the first 500 min of the study.

The release of MCPA from the hybrid nanoarchitectures was evaluated in in vitro tests in deionized water (pH approx. 5.5), simulating the conditions of rain. The kinetics of the release depends on the nanoarchitecture composition (Figure 7), but in all cases an initial fast release is observed, followed by another zone showing slower kinetics. These two regimes could be due to the initial release of more accessible MCPA, most likely related to interparticle diffusion, while the second zone could be due to diffusion of the intercalated herbicide molecules. The MCPA-LDH system showed the slowest release of MCPA, with around 35% lixiviated from the inorganic host after 8 h, being this value similar to those found for the release from MCPA_ie_-LDH [49]. Other studies reported a complete release of the herbicide in a similar time [50]. In contrast, the release from the MCPA_ie_-LDH/Sep1:1_150C hybrid nanoarchitecture, where the MCPA was incorporated by ion exchange showed a very rapid release, with practically 75% of the MCPA leached after the first 8 h. This result clearly confirms that the presence of the LDH as small nanoparticles attached to the fibrous clay may favor a rapid release of the intercalated species. In the hybrid nanoarchitectures prepared by coprecipitation and the same LDH/sepiolite ratio the release is slower. The slowest release occurred from the nanoarchitecture consolidated at 60 °C. A similar trend was observed when comparing the release from coprecipitated hybrid nanoarchitectures of other compositions consolidated at 60 and 150 °C (Figure S4, Supporting Information File 1). There is no clear explanation yet for this behavior. It might be ascribed to the different degree of hydration or the presence of OH^−^ species in the systems consolidated at lower temperature, which determines a different mechanism of attack of H^+^ to produce the degradation of the LDH and the subsequent release of entrapped MCPA. The fastest kinetics is observed with the lowest LDH content (Figure 7 and Figure S4, Supporting Information File 1). This behavior is probably related to the fact that size and aggregation state of the LDH nanoparticles increase with the LDH content in the nanoarchitecture, slowing down the kinetics of the process. The measured release after 8 h of contact with water varies with values of around 50% for nanoarchitectures consolidated at 60 °C (e.g., 43 and 51% for the 1:1 and 0.5:1 LDH/Sep nanoarchitectures, respectively) to around 70% for nanoarchitectures consolidated at 150 °C (e.g., 73% for MCPA-LDH/Sep1.1_150C, Figure 7B). After 8 h the release evolves differently towards a steady state, and after 48 h only the MCPA-LDH/Sep0.5:1_60C system completely released MCPA. These results confirm that the release of the herbicide from the hybrid nanoarchitectures may be tuned by selecting the specific composition and characteristics of the system, which makes them of interest for agricultural purposes.

Given that the amount of initial release of MCPA in all the formulations is quite high, the encapsulation of the hybrid nanoarchitectures in a protective biopolymer matrix was proposed to afford a better control over the release of the herbicide. In a previous study [49], a biopolymer mixture of alginate and zein incorporating the MCPA_ie_-LDH hybrid prepared by ion exchange was able to reduce the initial release of MCPA by approximately 10–15% in the first 8 h. In the current work, the MCPA-LDH/Sep0.5:1_60C nanoarchitecture was selected, as it releases 100% of the herbicide after a period of 48 h. The hybrid was dispersed in an alginate/zein matrix, with 17% of zein with respect to the total biopolymer mass. The mixture was added dropwise to a CaCl_2_ solution to produce bionanocomposite beads [36,49]. The encapsulation efficiency of the prepared bionanocomposite material was 51.2%, similar to that of other release systems based on the same encapsulation matrix [36].

**Figure 8:**
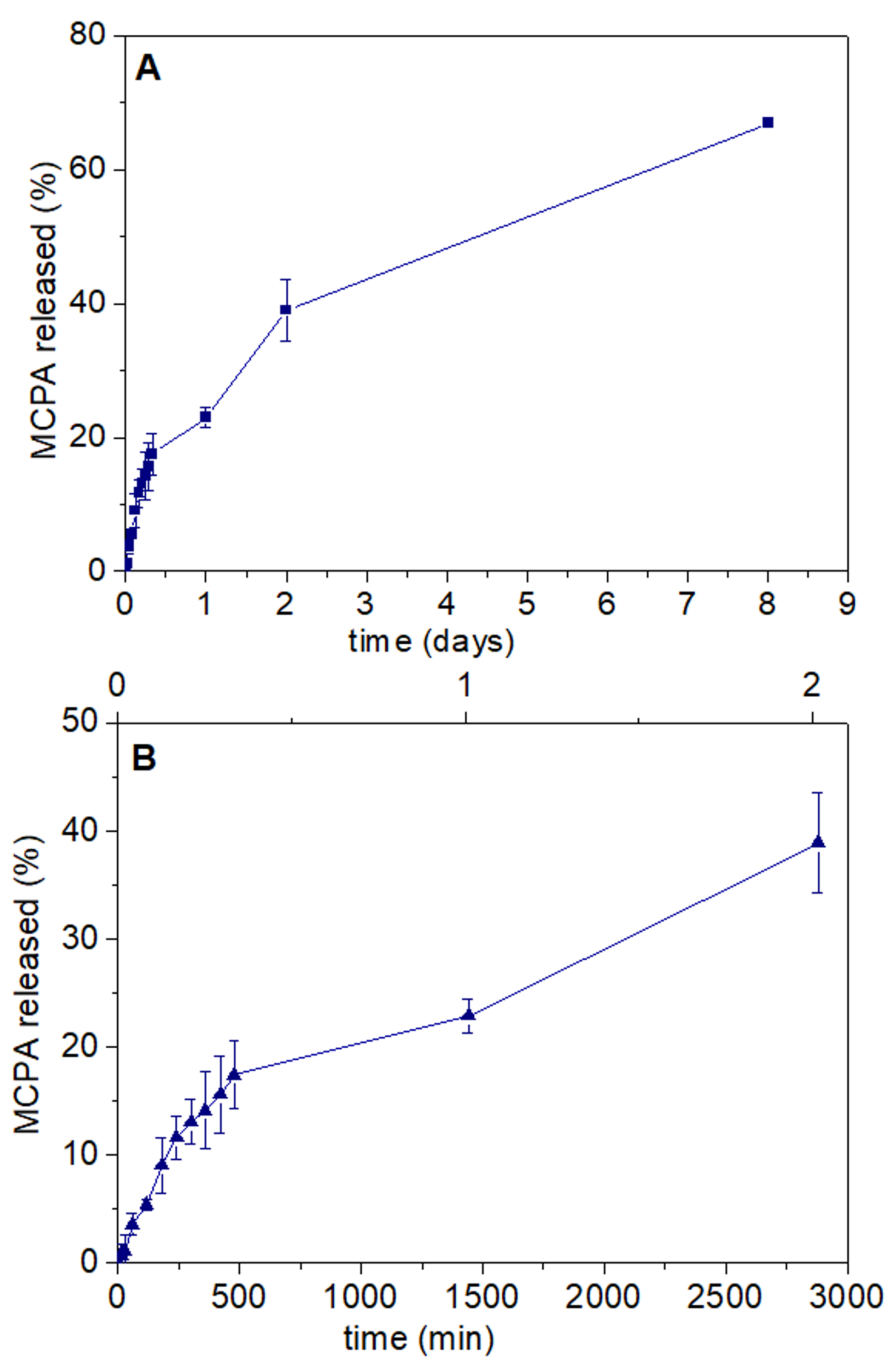
(A) In vitro release of MCPA encapsulated in the A-Z@MCPA-LDH/Sep bionanocomposite system over a period of 8 days in deionized water (pH 5.5), and (B) zoom of the same graph in the first 48 h of release.

In the bionanocomposite beads, the hydrophilicity of alginate is reduced by the presence of zein, contributing to a better control over the herbicide release. Figure 8 shows that release of MCPA from the A-Z@MCPA-LDH/Sep system is slower in the first 8 h than release from non-encapsulated systems, reaching approx. 40% after 48 h. A continuous study of the A-Z@MCPA-LDH/Sep0.5:1_60C formulation over 8 days was carried out, showing a release close to 70% in the presence of the biopolymer matrix. This result suggests that the bionanocomposite could reach 100% of MCPA release after about two weeks.

## Conclusion

This work reports on two procedures to prepare hybrid LDH/sepiolite nanoarchitectonic materials in which the herbicide MCPA is intercalated in the inorganic layered compound. The stability of the prepared MgAl-LDH/sepiolite nanoarchitectures allows for the ion exchange of interlayer anions by the anionic MCPA species. Moreover, it is possible to produce hybrid MCPA-LDH/sepiolite nanoarchitectures in a single coprecipitation step. This last approach allows for the incorporation of higher amounts of MCPA than the ion exchange reaction with the additional advantage of being less time-consuming. FTIR and ^29^Si NMR spectroscopic analysis corroborated that the LDH particles in the coprecipitated hybrid nanoarchitecture are chemically linked to the silanol groups that cover the silicate fibers, producing stable systems even using consolidation temperatures as low as 60 °C. The developed hybrid nanoarchitectures have been tested in vitro as systems for the controlled release of the incorporated organic species MCPA. In vitro tests carried out in deionized water showed that the herbicide release kinetics depended on the nanoarchitecture composition and the method of preparation. The materials with higher LHD content showed slower release rates. The herbicide could be completely released from the hybrid nanoarchitectures, confirming their suitability for the controlled release of pesticides in agriculture. To better control the release process, the hybrid nanoarchitectures can be encapsulated in a protective biopolymer matrix, such as alginate–zein, which delays the complete release up to several weeks. The presence of sepiolite in the hybrid nanoarchitectures could associate other active species to the formulation, profiting from the high capacity of this clay to adsorb numerous types of molecules. Finally, it is worthy to mention that the coprecipitation method opens the way to the production of other hybrid systems incorporating diverse organic and polymeric anionic species associated with nanometric LDH particles for controlled drug delivery and other applications.

## Experimental

### Starting reagents and materials

4-Chloro-2-methylphenoxyacetic acid (MCPA) was purchased from Sigma-Aldrich (*M*_W_ 200.62 g·mol^−1^, 97% purity). Sepiolite from Vallecas-Vicálvaro (Spain) was provided by TOLSA S.A. as Pangel^®^ S9, a commercial product of rheological grade that contains more than 95% pure sepiolite. Zein (Z) from maize, and alginate (A) were purchased from Sigma-Aldrich. Absolute ethanol was supplied by Panreac. Aqueous solutions were prepared from chemicals of analytical reagent grade: AlCl_3_·6H_2_O (>99%, Fluka), MgCl_2_·6H_2_O (99%, Carlo Erba), NaOH (≥98%, Fluka), ZnCl_2_ (>98%, Fluka). Deionized water (resistivity = 18.2 MΩ·cm) was obtained with a Maxima Ultrapure Water from Elga.

### Preparation of MCPA-LDH/sepiolite nanoarchitectures

MgAl-LDH/sepiolite (LDH/Sep) nanoarchitectures were prepared following the protocol described elsewhere [31]. In brief, a solution of MgCl_2_ and AlCl_3_ (9.34 mmol and 4.68 mmol) was drop-wise added to a dispersion of 4 g of sepiolite in 350 mL of deionized water at a rate of 2 mL/min, while kept under N_2_ flux to assure the removal of CO_2_. At the same time, a solution of 1 M NaOH was added with the 800 Dosino automatic dispenser from Metrohm in order to maintain the pH value constant at 9. After the addition of the salts to reach LDH/sepiolite products with 1:1, 0.5:1 or 0.3:1 theoretical weight ratio, the system was kept under magnetic stirring under a N_2_ flux for 24 h. The resulting LDH/Sep products were recovered by centrifugation, washed three times with deionized water, and dried at 150 °C under N_2_ flux (100 mL/min) for 3 h to consolidate the nanoarchitectures. For comparison, a LDH solid was prepared in the same way but without the presence of sepiolite. The intercalation of MCPA by ion exchange was performed using a solution of the herbicide prepared by dissolving 1.5 g of MCPA in 125 mL of ionized water and adjusting its pH value to 7 with 1 M NaOH to assure the presence of the organic molecule as an anion. This solution was slowly added to a dispersion prepared with 0.5 g of the selected LDH/Sep nanoarchitecture, or the LDH, in 125 mL of deionized water, with a final pH of approximately 9. The system was then kept under magnetic stirring and N_2_ flux for 72 h at room temperature. Subsequently, the solid was recovered by centrifugation, washed three times with water and dried at 60 °C overnight. The resulting materials were labeled as MCPA_ie_-LDH, MCPA_ie_-LDH/Sep1:1_150C, MCPA_ie_-LDH/Sep0.5:1_150C and MCPA_ie_-LDH/Sep0.3:1_150C.

In the same way, MCPA-LDH/sepiolite hybrid nanoarchitectures were prepared in one step by coprecipitation of the MgAl-LDH in presence of both sepiolite and MCPA. LDH formed on the surface of sepiolite fibers with intercalated herbicide anions instead of Cl^−^ ions. To this end, 4 g of sepiolite and 2.5 g of MCPA were dissolved in 350 mL of decarbonated deionized water. Again, the solution of MgCl_2_ and AlCl_3_ was varied in order to obtain hybrid nanoarchitectures with 2:1, 1:1, 0.5:1 and 0.3:1 theoretical LDH/Sep weight ratio. After the addition of the salts, the system was kept under magnetic stirring and N_2_ flux for 24 h. The solid was washed and recovered by centrifugation and then subjected to a controlled heat treatment at 60 or 150 °C for 3 h under air flow (100 mL/min) to consolidate the nanoarchitectures prepared from MCPA-LDH/Sep. The hybrid nanoarchitectures were labeled as MCPA-LDH/Sep2:1_60, MCPA-LDH/Sep2:1_150C, MCPA-LDH/Sep1:1_60C, MCPA-LDH/Sep1:1_150C, MCPA-LDH/Sep0.5:1_60C and MCPA-LDH/Sep0.5:1_150C, MCPA-LDH/Sep0.3:1_60C and MCPA-LDH/Sep0.3:1_150C. Following a similar protocol, the LDH was also coprecipitated in the presence of only MCPA to produce the corresponding MCPA-LDH intercalated material, which in this case was dried at 60 °C.

### Preparation of alginate–zein bionanocomposite beads

Alginate/zein beads were prepared following the following procedure adapted from Alcântara and co-workers [36]: i) The required amount of alginate to achieve a final total concentration of 2% in biopolymers was dissolved in 83 mL of water previously heated at 60 °C; ii) the required amount of zein (17% of the total biopolymer mass) and 34 mg of MCPA or the required amount of the MCPA-LDH or MCPA-LDH/Sep hybrids containing 34 mg of MCPA were incorporated into 20 mL of ethanol–water (80%,v/v); iii) the mixture was homogenized, and then slowly added to an alginate solution under magnetic stirring for approximately 30 min; iv) the formed gel was poured with a burette into a 5% CaCl_2_ solution to form the bionanocomposite beads, which were kept under constant stirring for 15 min. At the end of the process, the beads were washed with deionized water to remove residual Ca^2+^ ions and finally dried at 40 °C overnight. In this way, the following alginate–zein (A-Z) bionanocomposite beads were prepared: A-Z@MCPA-LDH and A-Z@MCPA-LDH/Sep0.5:1_60C, incorporating the intercalation compound or the hybrid nanoarchitecture, respectively.

### Characterization

Powder X-ray diffraction (XRD) data were collected on a Bruker D8 Advance diffractometer using a Cu Kα source, with a 2θ scan step of 2°·min^−1^ between 2 and 70°. The amount of the MCPA herbicide incorporated into the MCPA-LDH intercalation compounds and the MCPA-LDH/Sep hybrid nanoarchitectures was determined by CHN elemental chemical analysis using a LECO-CHNS-932 analyzer. Fourier transform infrared spectra (FTIR) were recorded from 4000 to 400 cm^−1^ with 2 cm^−1^ resolution in a Bruker IFS 66V-S spectrometer. Samples were prepared as pellets diluted in KBr or as pure samples pressed to form a tablet. ^29^Si solid-state MAS spectroscopy at 79.49 MHz was carried out on a BRUKER AV-400-W spectrometer equipped with a 4 mm MAS NMR probe, with the samples rotating at a rate of approximately 10 kHz and using a π/2 pulse of recycle delay of 5.9 μs and 5.0 s. Chemical shifts are referenced to tetramethylsilane (TMS) at δ = 0 ppm. Surface morphology of the samples was studied with the field-emision scanning electronic microscope (FE-SEM) FEI-NOVA NanoSEM 230, and TEM images were performed on a JEOL 2100F STEM 200 kV microscope.

### Release of MCPA in water

The release of MCPA from the MCPA-LDH hybrid, MCPA-LDH/Sep nanoarchitectures and the A-Z bionanocomposite materials was performed in 100 mL deionized water at pH 5.5, with the addition of the required quantity of material to provide 20 mg of MCPA. The experiment was maintained at room temperature under slow magnetic stirring. At predetermined times, aliquots of 3 mL were analyzed and evaluated by UV spectrophotometry at 279 nm [51] to determine the concentration of the released herbicide. After the analysis, the collected solution was returned to the initial solution to keep the volume constant. All experiments were performed in triplicate.

## Supporting Information

File 1 Additional experimental data.
