# Supplementary material for: Layered double hydroxide/sepiolite hybrid nanoarchitectures for the controlled release of herbicides
Source: Beilstein J Nanotechnol. 2019 Aug 9;10:1679–90. doi: 10.3762/bjnano.10.163 (PMC6693401; doi:10.3762/bjnano.10.163)
Supplement: File 1 — Additional experimental data. [file Beilstein_J_Nanotechnol-10-1679-s001.pdf]

BEILSTEIN JOURNAL OF NANOTECHNOLOGY

Supporting Information

for

Layered double hydroxide/sepiolite hybrid nanoarchitectures for the controlled release of herbicides

Ediana Paula Rebitski, Margarita Darder and Pilar Aranda

Beilstein J. Nanotechnol. **2019,** *10,* 1679–1690. doi:10.3762/bjnano.10.163

Additional experimental data

License and Terms: This is a supporting information file under the terms of the Creative Commons Attribution License (http://creativecommons.org/licenses/by/4.0). Please note that the reuse, redistribution and reproduction in particular requires that the authors and source are credited.
The license is subject to the *Beilstein Journal of Nanotechnology* terms and conditions: (https://www.beilstein-journals.org/bjnano)

Adsorption isotherm of MCPA in sepiolite

The adsorption isotherm of MCPA in sepiolite was carried out by adding a certain amount of sepiolite (300 mg) in water, and subsequently, variable amounts of MCPA were slowly added to the dispersion under continuous magnetic stirring, maintaining the system at room temperature for 48 hours. The supernatant was recovered by centrifugation and analyzed by UV spectrophotometry at 279 nm.

Figure S1 shows the adsorption isotherm of MCPA, showing that the maximum adsorbed amount of MCPA in sepiolite reached at the plateau is around 100 mg of MCPA / g of sepiolite.

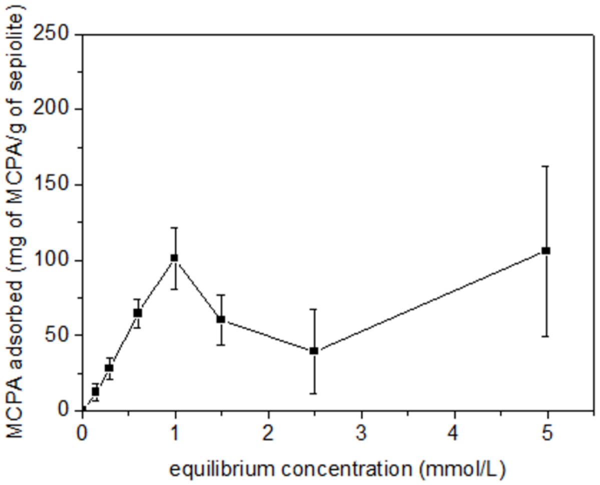

Figure S1: Adsorption isotherm at 298 K of MCPA (water solutions) in sepiolite.

FESEM and TEM images

The morphology of LDH and MCPA$_{ie}$-LDH and MCPA-LDH hybrid materials was analyzed by field emission scanning eletron microscopy (FESEM) and electron transmission microscopy (TEM) (Figure S2). Figure S2A shows that LDH exhibits a morphology of lamellar particles forming a *sandrose* structure [1]. According to Figure S2B and C, the incorporation of MCPA into MgAl-LDH, by ion-exchange and co-precipitation, gives rise to a more spongy morphology, where the nanolamines seem to be more separated. The TEM images of the materials show a morphology of round discs and some of them present an hexagonal form, typical of hydrotalcite materials (Figure S2B, S2D and S2F) [2,3].

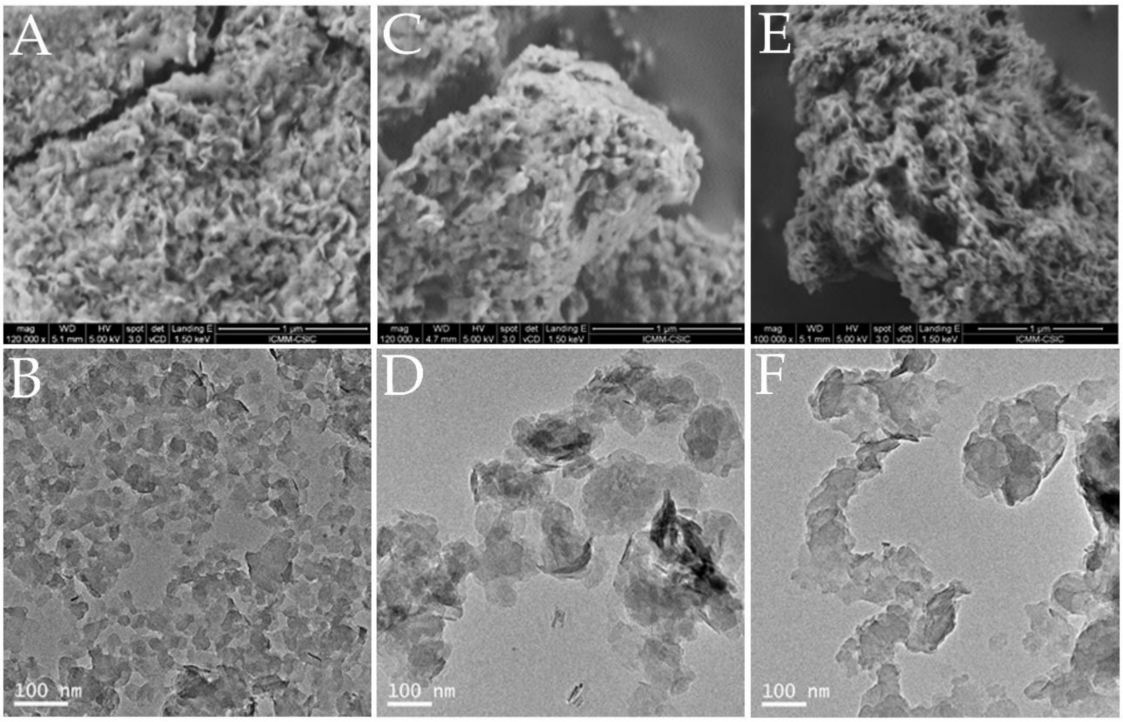

Figure S2: FESEM (upper row) and TEM (bottom row) images of the (A, B) LDH, (C, D) MCPA$_{ie}$-LDH and (E, F) MCPA-LDH (co-precipitation) hybrids.

Adsorption of MCPA on sepiolite in the presence of Mg and Al salts

Table S1 summarizes the mixtures of sepiolite, MCPA and the Mg and Al salts, in the same proportions than the hybrid nanoarchitectures, which were prepared to determine the uptake of MCPA on sepiolite under these conditions. The materials were kept under stirring for approximately 18 hours. The solid was recovered by centrifugation, washed 3 times with water and dried at 60 °C. The amount of adsorbed MCPA was determined by CHN and expressed as amount of MCPA per 100 grams of total material (not referred to the amount of sepiolite or salts added). In this case, the amount of MCPA incorporated by the effect of co-adsorption of MCPA with the Mg and Al salts does not seem to be dependent on the MCPA:salts ratio.

Table S1: Amount of MCPA adsorbed on sepiolite in the presence of Mg and Al salts.

**Samples**	**g MCPA/100g of material**	**mEq MCPA/100g of material**
Sep/MgAl-MCPA1:1	59.3	295.3
Sep/MgAl-MCPA0.5:1	54.7	272.5
Sep/MgAl-MCPA0.3:1	44.8	222.9

FTIR spectroscopy

The interactions of the MCPA herbicide in LDH or in the MCPA-LDH/Sep hybrid nanoarchitectures prepared by the coprecipitation method were studied by infrared spectroscopy (Figure S3). The main characteristic that confirms the presence of MCPA in the different prepared materials is the presence of the C=O stretching vibration of the MCPA protonated groups that appear between 1745 and 1600 cm^{-1}. This result confirms the presence of the herbicide as anionic species in the MCPA-LDH and in the nanoarchitectures. Also the presence of the

strong absorption band at approximately 1610 cm^{-1}, which can be assigned to the symmetric and asymmetric stretching vibration of the ionized COO^- groups, which may be overlapping with the 1630 cm^{-1} band of MgAl-LDH referring to the $\delta_{H\text{-}O\text{-}H}$ vibration mode of water molecules within the layers of inorganic solid [4–8]. The bands that appear in the low frequency region of the spectrum in the prepared hybrid nanoarchitectures are related to the vibration modes of the bands of both inorganic solids used.

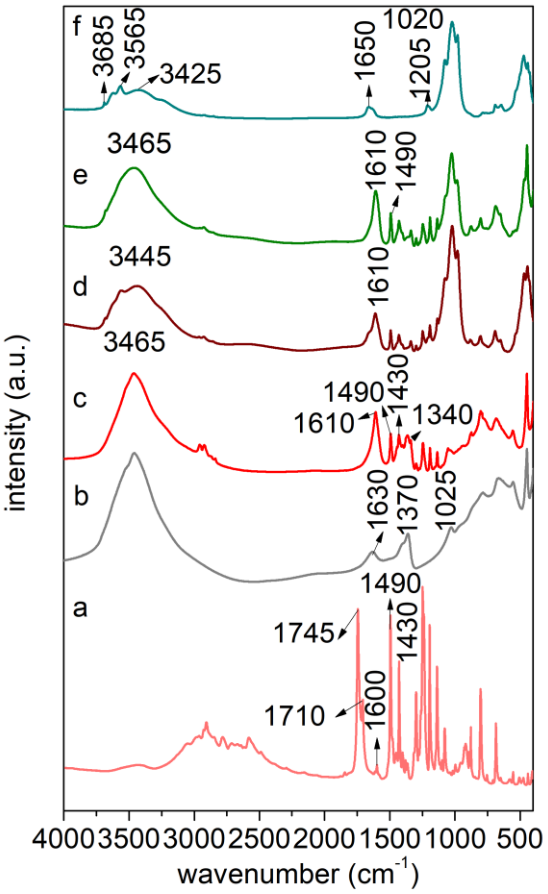

Figure S3: FTIR of the spectral region from 4000 to 400 cm^{-1} of (a) MCPA, (b) LDH, (c) MCPA-LDH, the (d) MCPA-LDH/Sep0.5:1_60 and (e) MCPA-LDH/Sep2:1_60C nanoarchitecture hybrids and (f) pristine sepiolite.

MCPA release tests

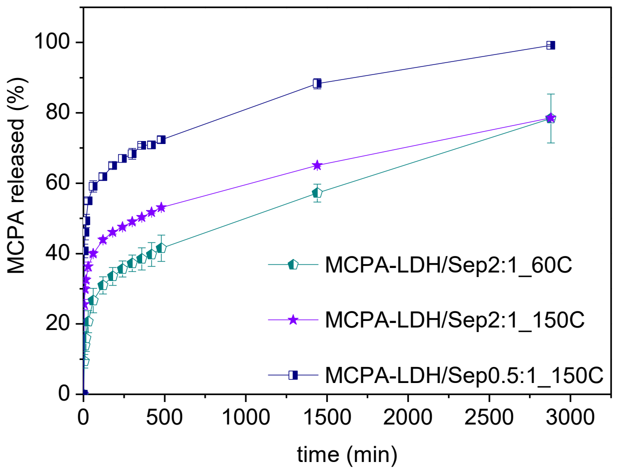

Figure S4: *In vitro* release of MCPA from the hybrid formulations in bidistilled water.

References

1. Leroux, F.; Gachon, J.; Besse, J.-P. J. *Solid State Chem.* **2004**, *177*, 245–250.

2. Costantino, U.; Marmottini, F.; Nocchetti, M.; Vivani, R. *Eur. J. Inorg. Chem* **1998**, 10, 1439–1446.

3. Okamoto, K.; Iyi, N.; Sasaki, T. *Appl. Clay Sci***.** **2007**, *37*, 23–31.

4. Casal, B.; Merino, J.; Serratosa, J.-M.; Ruiz-Hitzky, E.. *Appl. Clay Sci*. **2001**, *18*, 245–254.

5. Pavlovic, I.; Barriga, C.; Hermosín, M. C.; Cornejo, J.; Ulibarri, M. A. *Appl. Clay Sci*. **2005**, *30*, 125–133.

6. Cardoso, L. P.; Celis, R.; Cornejo, J.; Valim, J. B. *J. Agric. Food Chemistry*. **2006**, *54*, 5968–5975.

7. Bruna, F.; Celis, R.; Pavlovic, I.; Barriga, C.; Cornejo, J.; Ulibarri, M. A. *J. Hazard. Mater.* **2009**, *168*, 1476–1481.

8. Alromeed, A. A.; Scrano, L.; A. Bufo, S.; Undabeytia, T. *Pest Manag. Sci.* **2015**, *71*,1303–1310.
